# Development and Validation of a Prognostic Nomogram for Gastric Signet Ring Cell Carcinoma: A Multicenter Population-Based Study

**DOI:** 10.3389/fonc.2021.603031

**Published:** 2021-03-05

**Authors:** Shuairan Zhang, Yang Liu, Zihan Jiao, Zenan Li, Jin Wang, Ce Li, Xiujuan Qu, Ling Xu

**Affiliations:** ^1^ Department of Medical Oncology, The First Hospital of China Medical University, Shenyang, China; ^2^ Key Laboratory of Anticancer Drugs and Biotherapy of Liaoning Province, The First Hospital of China Medical University, Shenyang, China; ^3^ Liaoning Province Clinical Research Center for Cancer, Shenyang, China; ^4^ Key Laboratory of Precision Diagnosis and Treatment of Gastrointestinal Tumors, Ministry of Education, Shenyang, China

**Keywords:** gastric signet ring cell carcinoma, Surveillance Epidemiology and End Results (SEER), nomogram, prognosis, survival

## Abstract

**Background:**

Gastric signet ring cell carcinoma (GSRCC) is a rare disease associated with poor prognosis. A prognostic nomogram was developed and validated in this study to assess GSRCC patients’ overall survival (OS).

**Methods:**

Patients diagnosed with GSRCC from the Surveillance, Epidemiology, and End Results (SEER) database (2004–2016) and the First Hospital of China Medical University (CMU1h) were enrolled in this retrospective cohort study. Univariate and multivariate COX analysis was used to determine independent prognostic factors to construct the prognostic nomogram. Predictions were evaluated by the C-index and calibration curve. In addition, the receiver operating characteristic (ROC) curve, decision curve analysis (DCA), and Kaplan-Meier analysis were employed to assess the clinical utility of the survival prediction model.

**Results:**

Patients were classified into two cohorts. We randomly divided patients in the SEER database and CMU1h cohort into a training group (n=3068, 80%) and a validation group (n=764, 20%). Age, race, T stage, N stage, M stage, therapy, and tumor size were significantly associated with the prognosis of GSRCC patients. On this basis, a nomogram was constructed, with a C-index in the training and the validation cohorts at 0.772 (95% CI: 0.762–0.782) and 0.774 (95% CI: 0.752–0.796), respectively. The accuracy of the generated nomogram was verified through calibration plots. Similarly, compared with the traditional AJCC staging system, the results of the area under curve (AUC) calculated by ROC, DCA, and Kaplan-Meier curves, demonstrated a good predictive value of the constructed nomogram, compared to the traditional AJCC staging system.

**Conclusion:**

In the present study, seven independent prognostic factors of GSRCC were screened out. The established nomogram models based on seven variables provided a visualization of each prognostic factor’s risk and assisted clinicians in predicting the 1-, 3-, and 5-year OS of GSRCC.

## Introduction

Gastric cancer (GC) is a common malignancy, with a high mortality rate among all cancer types (1). Although GC incidence has declined in recent decades, GC from the diffuse type has an increasing incidence (2). According to Lauren’s classification, gastric signet ring cell carcinoma (GSRCC) is a diffuse GC type. Morphologically, it is characterized by prominent cytoplasmic mucin expression and an eccentrically localized, crescent-shaped nucleus (3). Compared with other GC types, GSRCC displays a unique biological behavior, usually at the advanced tumor stage and exhibits higher resistance to chemotherapy than non-SRCC (4). During the past decades, significant progress has been made in the diagnosis and treatment of GC, with the development of biologically targeted therapies and chemo- and radiotherapy.

Nevertheless, radical tumor resection (R0 resection) is still the optimal treatment (5). Due to the non-specific symptoms, such as pain or vomiting, curative resection is not suitable for most patients, resulting in a negative impact on GSRCC patients’ prognosis (6). Therefore, research on GC containing signet ring cell components becomes essential.

As stated by the American Joint Committee on Cancer (AJCC), the tumor lymph node metastasis (TNM) staging system has been widely used to predict the prognosis of cancer patients (7, 8). The 8th AJCC staging system for GC has been assessed by several large centers, providing excellent results in assessing the prognosis (9, 10). However, due to the association of non-TNM predictors, such as age, gender, race, tumor size, and treatment with GC patients’ survival, the AJCC staging system might not be useful in evaluating individual patient’s survival outcomes (11).

Nomograms are a novel, alternative model to evaluate patient prognosis and a statistical tool used in the evaluation of several cancer types (12, 13). Nomograms can estimate patients’ OS by integrating a variety of predictors into a single graphic representation. Compared with the traditional AJCC staging system, nomograms are significantly better at predicting individual risk. To the best of our knowledge, a prognostic nomogram for GSRCC patients has neither been developed nor validated.

Here, using the Surveillance, Epidemiology, and End Results (SEER) database (2004–2016) and the First Hospital of China Medical University (CMU1h) cohorts, a specific nomogram model for predicting the survival probability of GSRCC patients was developed and validated. We propose that our new model might serve as a tool for clinicians to better conduct risk assessments and improve patient management.

## Materials and Methods

### Patients

**Figure 1:**
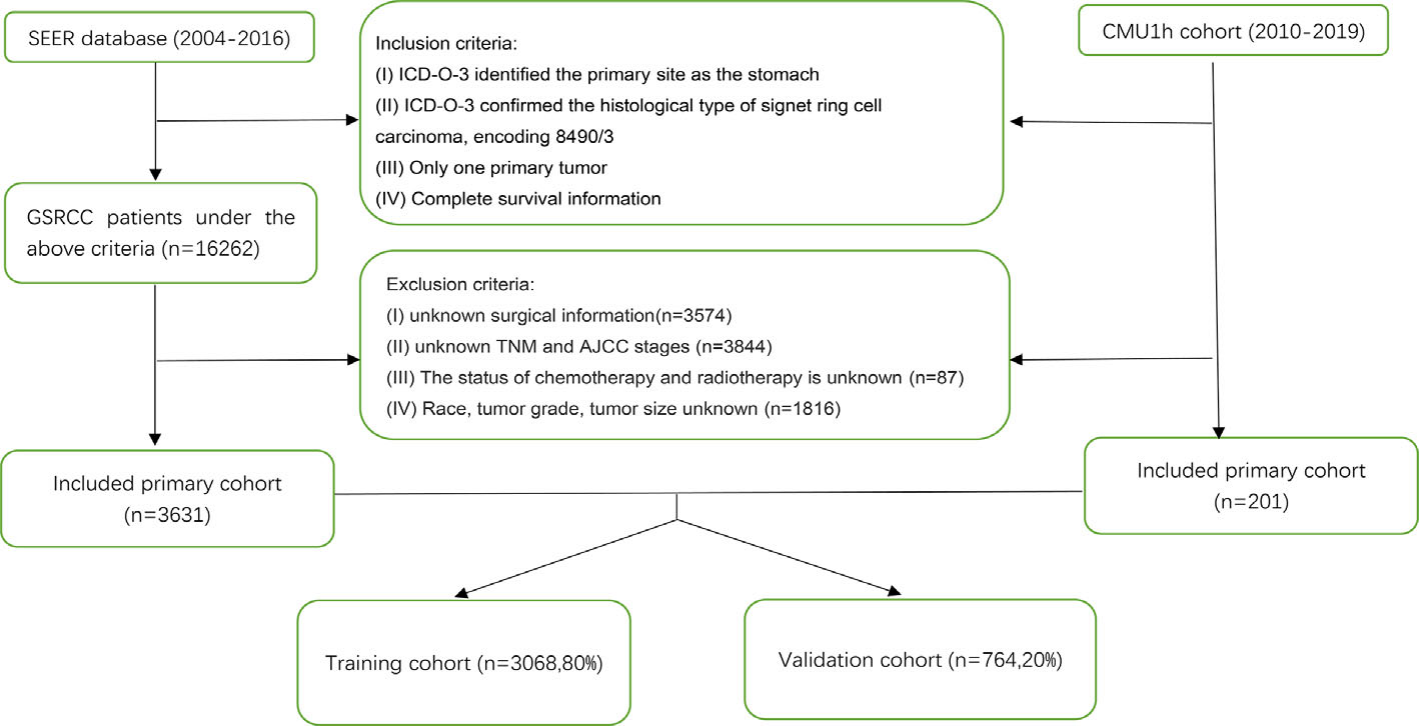
Flowchart of patients identified in this study.

We conducted a retrospective cohort study using the Surveillance, Epidemiology, and End Results (SEER) database of the National Cancer Institute (http://seer.cancer.gov/). The data of patients diagnosed from 2004 to 2016 were retrieved from the SEER 18 database using SEER*Stat, version 8.3.6. Patients diagnosed with GSRCC [histological diagnostic code 8390/3 in the International Classification of Diseases for Oncology, 3rd Edition (ICD-O-3)] from 2004 to 2016 were included in this study. Patients’ exclusion criteria were as follows: missing data concerning patients’ age, sex, race, tumor grade, AJCC stage, T stage, N stage, M stage, surgery, chemoradiotherapy, tumor size, and survival information. Patients with another primary tumor were also excluded. We only included patients who underwent radical gastrectomy and excluded patients who underwent other operations in the surgical variables. Furthermore, we included patients diagnosed with GSRCC between 2010 and 2019 from the First Hospital of China Medical University (CMU1h) according to the above-described criteria. The workflow of patient selection is shown in **Figure 1**. This study was performed following the Declaration of Helsinki and was approved by the institutional review board of the First Hospital of China Medical University.

### Clinical Variables

The following variables were selected as potential prognostic factors at the time of diagnosis (continuous variables were converted to categorical variables). Sixty was used as the cutoff value of age in this study, and the X-tile program was used to determine the cutoff point of tumor size: age (<60 years and ≥60 years), race (black, Caucasian, and others), gender (female, male), grade (G1, G2, G3, and G4), tumor staging according to the 8th Edition of AJCC System (stages I, II, III, IV, and unknown), T stage (T1, T2, T3, T4, and TX), N (N0, N1, N2, N3, and NX), M (M0, M1, and MX) stages, therapy (surgery plus chemotherapy/radiotherapy, surgery only, chemotherapy/radiotherapy only, none) and tumor size (<5 cm, ≥5 cm). OS was calculated from the date of diagnosis to the date of death or last follow-up. The median follow-up length was 16 [0–152.1] months. We performed re-staging in all the patients according to the 8th Edition of the AJCC Staging System.

### Statistical Analysis

We used the “caret” package in R version 4.0.2 to randomly group the patients, using 80% as the training cohort and the remaining 20% ​​as the validation cohort. We performed univariate COX proportional hazard regression analysis in a forward step-wise manner in the training group. Significant variables in univariate analysis (P<0.05) were carried into a multivariate COX analysis to obtain the hazard ratio (HR) and corresponding 95% confidential interval (CI) for every independent prognostic variable. Based on the proportional conversion of each regression coefficient in the multivariate analysis to a 0–100 point scale, we used the “rms” package to draw a nomogram. We further verified the internal and external accuracy of the nomogram. We used the Harrell consistency index (C-index) and calibration curves to evaluate the discrimination of the nomogram. Receiver operating characteristics (ROC) curves and decision curve analysis (DCA) were performed to show the nomogram’s clinical utility. According to the risk score of each patient calculated by the nomogram, we divided the patients into two different risk groups (low and high). Kaplan-Meier plots were constructed to analyze potential differences in patient overall survival between the high- and low-risk groups. All statistical analyses were performed using R software (version 4.0.2) (https://www.r-project.org/). A two-tailed P<0.05 was considered statistically significant.

## Results

### Patients Clinical Characterization

We identified 3,832 GSRCC cases from the SEER database and the CMU1h cohort, of which 3,068 patients were used as the training cohort, and the remaining 764 cases were used as the validation cohort (**Figure 1**). From the entire cohort of the selected patients, 2061 (53.8%) were >60 years old, 2005 (52.3%) were female patients, and 2295 (59.9%) were Caucasian, with the remaining being black or other (American Indians/AK natives, Asian/Pacific Islanders). The majority of patients exhibited poorly differentiated tumors (grade III/IV). Concerning the AJCC staging, 1246 (32.6%) patients presented with stage IV tumors. As for TNM staging, a portion of the patient was classified as T2 (35.7%), N0 (35.9%), or N1 (30.2%), and M0 (76.2%). In terms of treatment, 1911 (49.9%) patients had received radical surgery plus chemotherapy or radiotherapy. A total of 2,062 (53.8%) patients presented with tumors <5 cm at diagnosis. **Table 1** presents the patients’ clinicopathological characteristics.

**Table 1:** Baseline clinicopathological characteristics and treatment regimens of patients.

Variables	All patients, n (%)	Training set, n (%)	Validation set, n (%)	
Total	3,832 (100.0)	3,068 (80.0)		764 (20.0)
Age				
<60	1,771 (46.2)	1,413 (46.1)		358 (46.9)
≥60	2,061 (53.8)	1,655 (53.9)		406 (53.1)
Race				
Black	495 (12.9)	403 (13.1)		92 (12.0)
White	2,295 (59.9)	1,845 (60.1)		450 (58.9)
Others	1,042 (27.2)	820 (21.4)		222 (29.1)
Sex				
Female	2,005 (52.3)	1,610 (52.5)		395 (51.7)
Male	1,827 (47.7)	1,458 (47.5)		369 (48.3)
Grade				
G1	11 (0.2)	8 (0.2)		3 (0.3)
G2	104 (2.7)	82 (2.7)		22 (2.9)
G3	3,604 (94.1)	2,882 (93.9)		722 (94.5)
G4	113 (3.0)	96 (3.1)		17 (2.2)
AJCC stage				
I	1,001 (26.2)	828 (27.0)		177 (23.2)
II	611 (16.0)	485 (15.8)		128 (16.8)
III	862 (22.6)	682 (22.2)		180 (23.6)
IV	1,246 (32.6)	994 (32.4)		256 (33.5)
Unknown stage	102 (2.7)	79 (2.8)		23 (3.0)
T stage				
T1	727 (19.0)	597 (19.5)		130 (17.0)
T2	1,368 (35.7)	1,109 (36.1)		259 (33.9)
T3	982 (25.6)	763 (24.9)		219 (28.7)
T4	548 (14.3)	444 (14.5)		104 (13.6)
TX	207 (5.4)	155 (5.0)		52 (6.8)
N stage				
N0	1,374 (35.9)	1,116 (36.4)		258 (33.8)
N1	1,159 (30.2)	920 (30.0)		239 (31.3)
N2	739 (19.3)	594 (19.4)		145 (19.0)
N3	422 (11.0)	332 (10.8)		90 (11.8)
NX	138 (3.6)	106 (3.5)		32 (4.2)
M stage				
M0	2,921 (76.2)	2,359 (76.9)		562 (73.6)
M1	844 (22.0)	659 (21.5)		185 (24.2)
MX	67 (1.8)	50 (1.6)		17 (2.2)
Therapy				
None	265 (6.9)	204 (6.6)		61 (8.8)
Surgery only	1,241 (32.4)	994 (32.4)		247 (32.3)
Chemo/Radio only	415 (10.8)	336 (11.0)		79 (10.3)
Surgery plus Chemo/Radio	1,911 (49.9)	1,534 (50.0)		377 (49.3)
Tumor size				
<5cm	2,062 (53.8)	1,661 (54.1)		401 (52.5)
≥5cm	1,770 (46.2)	1,407 (45.9)		363 (47.5)

### Construction of the Nomogram

**Figure 2:**
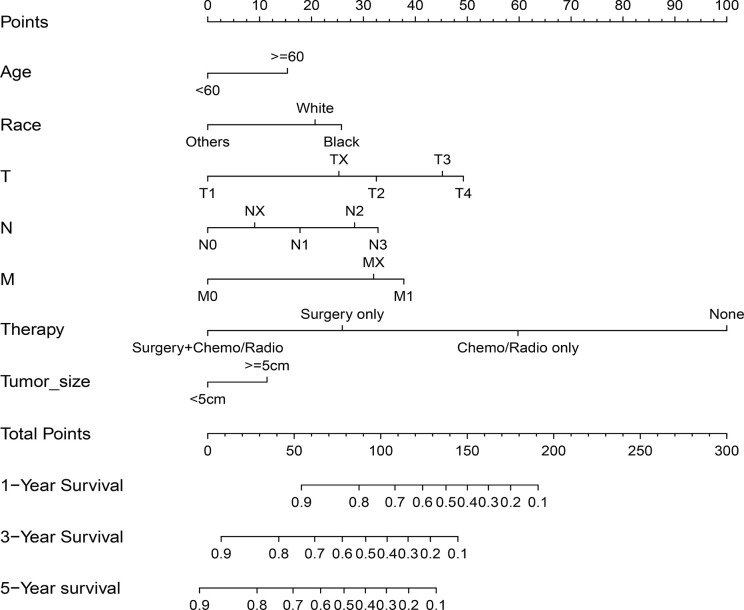
Nomogram predicting 1-, 3-, and 5-year overall survival (OS) for patients with gastric signet ring cell carcinoma (GSRCC). The total points are calculated by summing up the points for each factor. The total points correspond to the patient’s 1-, 3-, and 5-year survival probability.

Age, race, AJCC stage, T stage, N stage, M stage, treatment method, and tumor size were confirmed to be closely related to the patient’s OS (P<0.05) in univariate and multivariate analysis in the training cohort. **Table 2** lists the risk ratio of each variable to OS in the univariate and multivariate COX risk models. Since the AJCC stage is a comprehensive variable for T, N, and M stages, we did not include the variable AJCC staging in the nomogram. Based on the seven variables identified in the previous multivariate COX proportional hazard model, we established a nomogram to predict 1-, 3-, and 5-year OS in patients with GSRCC (**Figure 2**). The score of each variable was obtained by establishing a vertical upward line; the scores obtained by each variable could then be summed to achieve the total score. A vertical downward line denotes the specific probability of 1-, 3-, and 5-year survival rates of GSRCC patients.

**Table 2:** Risk factors affecting patients’ overall survival (OS), according to the univariate and multivariate Cox analysis.

Variables	No. of patients		Univariate analysis	Multivariate analysis	
		P value		HR (95% CI)	P value
Age		<0.001			
<60	1,413			Reference	
≥60	1,655			1.41 (1.29–1.55)	<0.001
Race		<0.001			
Black	403			Reference	
White	1,845			0.89 (0.78–1.01)	0.09
Others	820			0.56 (0.48–0.66)	<0.001
Sex		0.900			
Female	1,610			–	–
Male	1,458			–	–
Grade		0.128			
G1	8			–	–
G2	82			–	–
G3	2,882			–	–
G4	96			–	–
AJCC stage		<0.001			
I	828			Reference	
II	485			1.85 (1.47–2.33)	<0.001
III	682			2.63 (2.04–3.40)	<0.001
IV	994			2.95 (2.18–3.99)	<0.001
UKN stage	79			1.92 (1.31–2.83)	<0.001
T stage		<0.001			
T1	597			Reference	
T2	1,109			2.08 (1.75–2.47)	<0.001
T3	763			2.77 (2.27–3.39)	<0.001
T4	444			3.04 (2.51–3.67)	<0.001
TX	155			1.77 (1.37–2.28)	<0.001
N stage		<0.001			
N0	1,116			Reference	
N1	920			1.49 (1.31–1.70)	<0.001
N2	594			1.89 (1.63–2.19)	<0.001
N3	332			2.10 (1.76–2.50)	<0.001
NX	106			1.23 (0.96–1.57)	0.107
M stage		<0.001			
M0	2,359			Reference	
M1	659			2.34 (2.08–2.64)	<0.001
MX	50			2.05 (1.48–2.84)	<0.001
Therapy		<0.001			
None	204			Reference	
Surgery only	994			0.19 (0.15–0.23)	<0.001
Chemo/Radio only	336			0.40 (0.33–0.49)	<0.001
Surgery plus Chemo/Radio	1,534			0.11 (0.09–0.13)	<0.001
Tumor size		<0.001			
<5cm	1,661			Reference	
≥5cm	1,407			1.29 (1.17–1.43)	<0.001

### Nomogram Calibration and Validation

**Figure 3:**
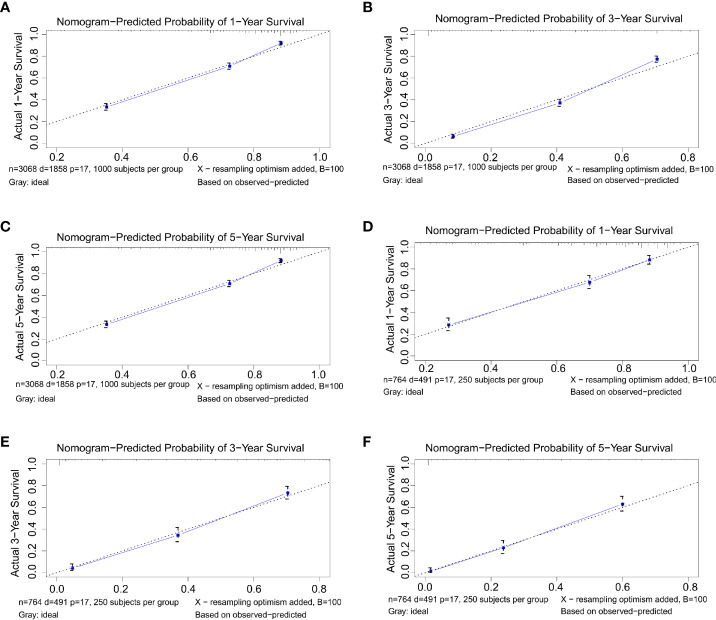
**(A–C)** Nomogram calibration plots to predict 1-, 3-, and 5-year overall survival (OS) in the training cohort. **(D–F)** Nomogram calibration plots to predict 1-, 3-, and 5-year OS in the validation cohort.

C-index and calibration curves of the nomogram were used to validate our model-training cohort validation. The predicted C-index by the nomogram concerning the training cohort was higher than that of the AJCC staging system, 0.772 vs. 0.701 (95% CI: 0.762–0.781 vs. 0.689–0.713, respectively) in the training cohort and 0.774 vs. 0.699 (95% CI: 0.752–0.796 vs. 0.675–0.723, respectively) in the validation cohort. Survival calibration plots showed excellent consistency between the nomogram-predicted survival probabilities and actual observation in the training cohort and the validation cohort **(** **Figure 3** **)**.

### Comparison of the Nomogram and AJCC Staging System

**Figure 4:**
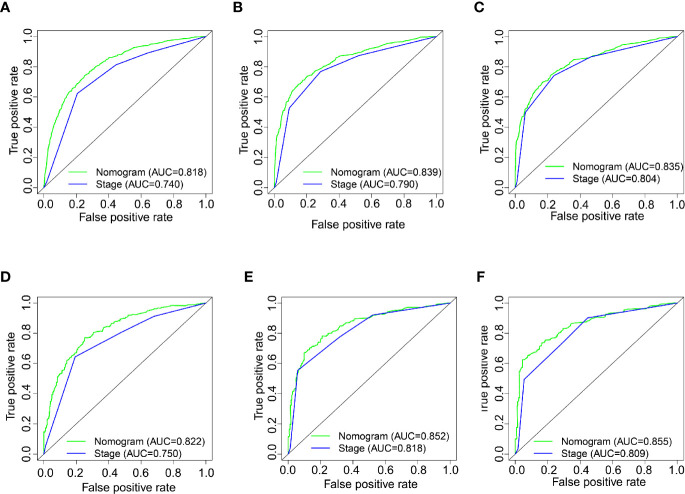
**(A–C)** Nomogram and AJCC staging system ROC curves for predicting 1-, 3-, and 5-year OS in the training cohort. **(D–F)** Nomogram and AJCC staging system ROC curves for predicting 1-, 3-, and 5-year OS in the validation cohort.

**Figure 5:**
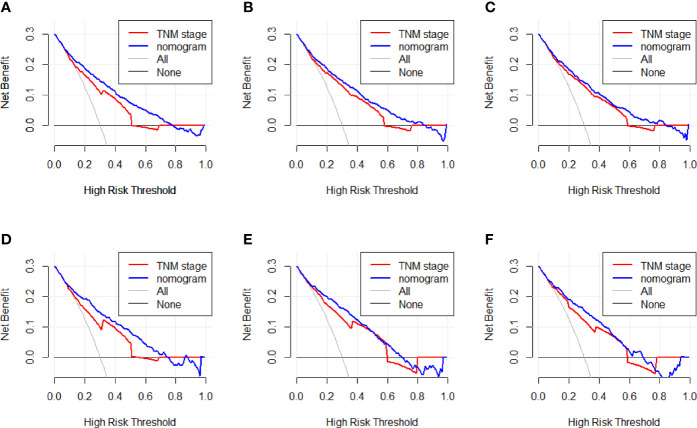
**(A–C)** Nomogram and AJCC staging system DCA analysis predicting 1-, 3-, and 5-year OS in the training cohort. **(D–F)** Nomogram and AJCC staging system DCA analysis predicting 1-, 3-, and 5-year OS in the validation cohort.

We established the ROC curve and calculated the corresponding AUC to compare the nomogram and AJCC staging accuracy in predicting patients’ overall survival. The 1-, 3-, and 5-year AUCs, corresponding to the training cohort predicted by ROC analysis of the nomogram, were 0.818, 0.839, and 0.835, respectively, whereas the AUC values calculated from the AJCC staging system were 0.740, 0.790, and 0.804 **(** **Figures 4A–C** **)**. The 1-, 3-, and 5-year AUC predicted by the nomogram in the validation cohort were 0.822, 0.852, and 0.855, respectively, higher than 0.750, 0.818, and 0.809 of the AJCC staging system **(** **Figures 4D–F** **)**. It means that the nomogram had superior predictive ability than the AJCC staging system. In addition, as shown in **Figure 5**, the DCA showed good performance of the nomogram in clinical use and is better than the traditional AJCC staging system.

### Survival Analysis

**Figure 6:**
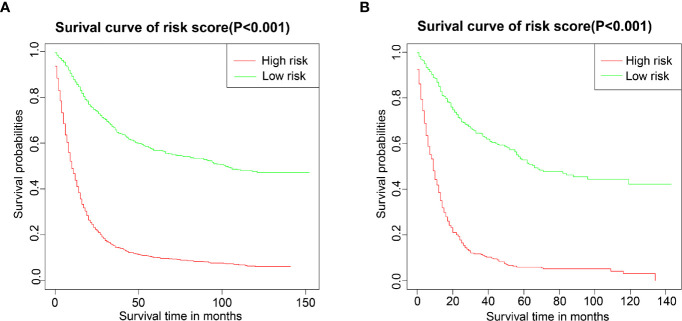
Overall survival (OS) Kaplan-Meier curves for patients in the low- and high-risk groups. **(A)** training group; **(B)** validation group.

We performed a survival analysis by Kaplan-Meier plots in the training and validation cohort. Patients from the training cohort exhibited OS ranging from 1 to 152.1 months, with a median of 17 months. Overall, 1-, 3-, and 5-year survival rates were 67.3%, 46.4%, and 41.9%, respectively. In the validation cohort, patients’ OS ranged from 1 to 152 months, with a median of 21 months. Overall, 1-, 3-, and 5-year survival rates were 63.7%, 43.9%, and 38.4%, respectively. Subsequently, the patients were divided into high- and low-risk groups based on the nomogram’s median score. Kaplan-Meier curves showed that the patients’ OS in the high-risk group was lower than that of patients from the low-risk group **(** **Figure 6** **)**, supporting the use of our generated nomogram in GRSCC patient stratification.

## Discussion

Gastric signet ring cell carcinoma (GSRCC) exhibits distinct tumorigenic properties and epidemiological distribution compared to other forms of gastric cancer (GC) (14, 15). The American Joint Committee on Cancer (AJCC) staging system is currently used to determine GC patients’ prognosis. However, current prediction models of GC monitoring are not suitable to monitor GSRCC. Notably, the AJCC staging system does not account for some significant clinicopathological characteristics, like age, gender, and treatment method related to patients’ survival. In this respect, our generated nomogram poses a significant advantage due to the possibility of integrating variables that are available and quantifiable to provide prognostic information (16). Although previous studies have compared the performance of nomograms to predict the prognosis of GC (17, 18), the nomogram we constructed can be better used to predict the prognosis of GSRCC.

Nomograms have been successfully established to predict the survival of patients with GC. A previous report described the construction of a nomogram combined with five clinicopathological features to predict the prognosis of GC with hepatitis B virus infection, which demonstrated a significant predictive value (19). Roberto et al. built a nomogram of postoperative survival probability of GC patients based on age, preoperative performance, lymph node invasion, presence of residual tumors, and depth of tumor invasion (20). Clinical data of patients undergoing radical gastrectomy (R0 resection) in three centers plus SEER database-derived patient data were retrospectively analyzed (21). The authors developed a simple nomogram to assess individual survival probability after R0 resection of GC tumors. In the present study, a large cohort was used to establish a novel nomogram for predicting the prognosis of GSRCC.

Consistent with the AJCC staging system, our newly generated nomogram showed a significant impact of infiltration depth and the presence of lymph node and distant metastasis in the prediction of survival outcome. In addition, age, race, whether a radical operation, chemotherapy, or radiotherapy was performed, and tumor size were identified as independent prognostic factors in the context of GSRCC. Using these variables as independent prognostic factors in the nomogram might increase the predictive power of the model. It has been reported that the prognosis of young GSRCC patients with low stage tumors, which underwent radical surgery, is better than other GSRCC patients in terms of survival (22). It is well established that older age has low survival time because older people usually have more comorbidities (23). On the contrary, younger patients have better physical and psychological conditions, leading to a better prognosis (24). In another study, compared with Asian races, black races had a higher risk of death from GSRCC (25). Several studies have found similar results. Wang et al. reported that Asian patients have better overall survival than Caucasians and African Americans (26). Surgical resection (R0) remains the only curative modality for localized gastric cancer (27). Furthermore, it was found that the survival rate of GSRCC patients who received radiotherapy after surgery was higher than that of patients who received surgery alone (28). Perioperative chemotherapy or postoperative (adjuvant) chemoradiotherapy can effectively improve patients’ overall survival rate (29, 30). Our results further support previous findings of larger tumor size as an independent prognostic factor negatively correlated with GSRCC patient survival. GSRCC patients with larger tumors might have a higher probability of invasive growth and lymph node metastasis (14, 31). In contrast, we found no significant correlation between histological grade or gender and patient survival. In the present study, approximately 97% of the samples were in the grade III/grade IV histological classification. Although in most tumors, the histological grade is one of the indicators that determine patients’ prognosis, since the vast majority of GSRCC patients exhibit high histological grades, histological grade failed to be a risk factor for determining patients’ prognosis in the present study. In addition, gender did not seem to be one of the risk factors for predicting the prognosis of GSRCC, consistent with the findings of Chon et al. (32).

The influence of the above factors on the prognosis of GSRCC might impair the traditional AJCC staging system’s prediction accuracy. Therefore, we developed a nomogram to predict the survival of GSRCC patients based on multiple prognostic factors. After internal and external verification, the nomogram showed good individualized risk prediction and stratification capacity. The C-index and calibration plots of the nomogram showed the model’s good discrimination and calibration features. Compared to the standard AJCC system, our nomogram performed better at predicting 1-, 3-, and 5-year OS accurately. In addition, DCA analysis revealed that our nomogram had better predictive ability compared with the AJCC staging system.

Our newly developed nomogram identified treatment method, tumor stage, and infiltration depth as the significant risk factors affecting the prognosis of GSRCC patients. Compared with the traditional AJCC staging system, the nomogram established based on more clinicopathological information and treatment conditions can more accurately evaluate and predict the prognosis of GSRCC. Therefore, it is expected to help clinicians to better identify risks for patients and make clinical decisions.

Despite the significant findings, we acknowledge the limitations of our study. First, as a large-scale retrospective study, patient selection might be affected by selection biases. Second, we did not include potentially important information, such as the specific location of the distant metastases and surgery methods (33, 34). All the factors we included are known risk factors, and there are many risk factors related to the prognosis of GSRCC that should be studied further. Despite such limitations, our prognostic nomogram was shown to be a useful and instructive model that accurately predicts the individual outcome of GSRCC patients.

## Conclusion

In summary, we generated a nomogram based on seven clinicopathological characteristics identified by univariate and multivariate COX analyses. The proposed nomogram can efficiently help clinicians predict the 1-, 3-, and 5-year OS of GSRCC patients. Furthermore, this nomogram might help stratify the risk and aid in clinical decision-making of GSRCC patients.

## Data Availability Statement

The original contributions presented in the study are included in the article/supplementary material. Further inquiries can be directed to the corresponding authors.

## Ethics Statement

Written informed consent was obtained from the individual(s) for the publication of any potentially identifiable images or data included in this article.

## Author Contributions

LX and XQ designed the project, reviewed, and edited the manuscript, respectively. SZ, YL, ZJ, and ZL performed the study selection, data extraction, statistical analyses, and wrote the main manuscripts. JW and CL contributed to the classification criteria discussion. All authors contributed to the article and approved the submitted version.

## Funding

This study was funded by the National Natural Science Foundation of China (No. 81673025, No.81902998), Science and Technology Youth Projects of the Education Department of Liaoning Province (QN2019004), and the National Key Research and Development Program of China (NO. 2017YFC1308900), and National Science and Technology Major Project of the Ministry of Science and Technology of China (No. 2017ZX09304025), and The Key Research and Development Program of Liaoning Province (2018225060).

## Conflict of Interest

The authors declare that the research was conducted in the absence of any commercial or financial relationships that could be construed as a potential conflict of interest.

## References

[1] Fitzmaurice C Allen C Barber RM Barregard L Bhutta ZA Brenner H. Global, Regional, and National Cancer Incidence, Mortality, Years of Life Lost, Years Lived With Disability, and Disability-Adjusted Life-years for 32 Cancer Groups, 1990 to 2015: A Systematic Analysis for the Global Burden of Disease Study. JAMA Oncol (2017) 3(4):524–48. 10.1001/jamaoncol.2016.5688 PMC6103527 27918777

[2] Siegel RL Miller KD Jemal A. Cancer statistics, 2019. CA Cancer J Clin (2019) 69(1):7–34. 10.3322/caac.21551 30620402

[3] Fléjou JF. [WHO Classification of digestive tumors: the fourth edition]. Ann Pathol (2011) 31(5 Suppl):S27–31. 10.1016/j.annpat.2011.08.001 22054452

[4] Piessen G Messager M Leteurtre E Jean-Pierre T Mariette C. Signet ring cell histology is an independent predictor of poor prognosis in gastric adenocarcinoma regardless of tumoral clinical presentation. Ann Surg (2009) 250(6):878–87. 10.1097/SLA.0b013e3181b21c7b 19855261

[5] Longmire WP Jr. Gastric carcinoma: is radical gastrectomy worth while? Ann R Coll Surg Engl (1980) 62(1):25–30. 7362184 PMC2492281

[6] Mengardo V Treppiedi E Bencivenga M Dal Cero M Giacopuzzi S. Tailored treatment for signet ring cell gastric cancer. Updates Surg (2018) 70(2):167–71. 10.1007/s13304-018-0550-4 29948660

[7] Bando E Makuuchi R Irino T Tanizawa Y Kawamura T Terashima M. Validation of the prognostic impact of the new tumor-node-metastasis clinical staging in patients with gastric cancer. Gastric Cancer (2019) 22(1):123–9. 10.1007/s10120-018-0799-9 29357013

[8] Son T Sun J Choi S Cho M Kwon IG Kim HI. Multi-institutional validation of the 8th AJCC TNM staging system for gastric cancer: Analysis of survival data from high-volume Eastern centers and the SEER database. J Surg Oncol (2019) 120(4):676–84. 10.1002/jso.25639 31338834

[9] In H Solsky I Palis B Langdon-Embry M Ajani J Sano T. Validation of the 8th Edition of the AJCC TNM Staging System for Gastric Cancer using the National Cancer Database. Ann Surg Oncol (2017) 24(12):3683–91. 10.1245/s10434-017-6078-x 28895113

[10] Graziosi L Marino E Donini A. Survival comparison in gastric cancer patients between 7th and 8th edition of the AJCC TNM staging system: The first western single center experience. Eur J Surg Oncol (2019) 45(6):1105–8. 10.1016/j.ejso.2018.12.010 30595468

[11] Guo J Yu J Xu Z Sun X Zheng J. The role of surgery in patients aged 85 years or older with resectable gastric cancer: a propensity score matching analysis of the SEER database. Scand J Gastroenterol (2020) 55(6):694–700. 10.1080/00365521.2020.1769175 32459113

[12] Balachandran VP Gonen M Smith JJ DeMatteo RP. Nomograms in oncology: more than meets the eye. Lancet Oncol (2015) 16(4):e173–80. 10.1016/S1470-2045(14)71116-7 PMC4465353 25846097

[13] Fu YP Ni XC Yi Y Cai XY He HW Wang JX. A Novel and Validated Inflammation-Based Score (IBS) Predicts Survival in Patients With Hepatocellular Carcinoma Following Curative Surgical Resection: A STROBE-Compliant Article. Medicine (2016) 95(7):e2784. 10.1097/MD.0000000000002784 26886627 PMC4998627

[14] Chen TH Lin WR Lee C Chiu CT Hsu JT Yeh TS. Prognostic Stratification of Advanced Gastric Signet Ring Cell Carcinoma by Clinicopathological Factors and GALNT14 Genotype. J Cancer (2018) 9(19):3540–7. 10.7150/jca.26293 PMC6171017 30310511

[15] Tian MM Zhao AL Li ZW Li JY. Phenotypic classification of gastric signet ring cell carcinoma and its relationship with clinicopathologic parameters and prognosis. World J Gastroenterol (2007) 13(23):3189–98. 10.3748/wjg.v13.i23.3189 PMC4436604 17589897

[16] Shu Y Zhang W Hou Q Zhao L Zhang S Zhou J. Prognostic significance of frequent CLDN18-ARHGAP26/6 fusion in gastric signet-ring cell cancer. Nat Commun (2018) 9(1):2447. 10.1038/s41467-018-04907-0 29961079 PMC6026495

[17] Yu C Zhang Y. Development and validation of prognostic nomogram for young patients with gastric cancer. Ann Trans Med (2019) 7(22):641–641. 10.21037/atm.2019.10.77 PMC6944578 31930042

[18] Du F Sun Z Jia J Yang Y Yu J Shi Y. Development and Validation of an Individualized Nomogram for Predicting Survival in Patients with Esophageal Carcinoma after Resection. J Cancer (2020) 11(14):4023. 10.7150/jca.40767 32368284 PMC7196250

[19] He Y Mao M Shi W He Z Zhang L Wang X. Development and validation of a prognostic nomogram in gastric cancer with hepatitis B virus infection. J Trans Med (2019) 17(1):98. 10.1186/s12967-019-1841-3 PMC6434786 30909980

[20] Roberto M Botticelli A Strigari L Ghidini M Onesti CE Ratti M. Prognosis of elderly gastric cancer patients after surgery: a nomogram to predict survival. Med Oncol (Northwood London England) (2018) 35(7):111. 10.1007/s12032-018-1166-8 29923032

[21] Zheng ZF Lu J Wang W Desiderio J Li P Xie JW. Development and External Validation of a Simplified Nomogram Predicting Individual Survival After R0 Resection for Gastric Cancer: An International, Multicenter Study. Ann Surg Oncol (2018) 25(8):2383–90. 10.1245/s10434-018-6551-1 29881929

[22] Lu M Yang Z Feng Q Yu M Zhang Y Mao C. The characteristics and prognostic value of signet ring cell histology in gastric cancer: A retrospective cohort study of 2199 consecutive patients. Medicine (2016) 95(27):e4052. 10.1097/MD.0000000000004052 27399088 PMC5058817

[23] Suh DD Oh ST Yook JH Kim B-S Kim BS. Differences in the prognosis of early gastric cancer according to sex and age. Ther Adv Gastroenterol (2017) 10(2):219–29. 10.1177/1756283X16681709 PMC5298480 28203280

[24] Zhang J Gan L Xu M Huang M Zhang X Gong Y. The prognostic value of age in non-metastatic gastric cancer after gastrectomy: a retrospective study in the US and China. J Cancer (2018) 9(7) :1188–99. 10.7150/jca.22085 PMC5907667 29675100

[25] Taghavi S Jayarajan SN Davey A Willis AI. Prognostic significance of signet ring gastric cancer. J Clin Oncol (2012) 30(28):3493–8. 10.1200/JCO.2012.42.6635 PMC3454770 22927530

[26] Wang A Squires III M H Melis M Poultsides GA Norton JA Jin LX. Stage-specific prognostic effect of race in patients with resectable gastric adenocarcinoma: an 8-institution study of the US gastric cancer collaborative. J Am Coll Surg (2016) 222(4):633–43. 10.1016/j.jamcollsurg.2015.12.043 26905187

[27] Schizas D Mastoraki A Naar L Tsilimigras DI Katsaros I Fragkiadaki V. Histone deacetylases (HDACs) in gastric cancer: an update of their emerging prognostic and therapeutic role. Curr Med Chem (2020) 27(36):6099–111. 10.2174/0929867326666190712160842 31309879

[28] Wei F Lyu H Wang S Chu Y Chen F. Postoperative Radiotherapy Improves Survival in Gastric Signet-Ring Cell Carcinoma: a SEER Database Analysis. J Gastric Cancer (2019) 19(4):393–407. 10.5230/jgc.2019.19.e36 31897342 PMC6928086

[29] Cunningham D Allum WH Stenning SP Thompson JN Van de Velde CJ Nicolson M. Perioperative chemotherapy versus surgery alone for resectable gastroesophageal cancer. N Engl J Med (2006) 355(1):11–20. 10.1056/NEJMoa055531 16822992

[30] Macdonald JS Smalley SR Benedetti J Thompson JN Van de Velde CJ Nicolson M. Chemoradiotherapy after surgery compared with surgery alone for adenocarcinoma of the stomach or gastroesophageal junction. N Engl J Med (2001) 345(10):725–30. 10.1056/NEJMoa010187 11547741

[31] Zhou L Li W Cai S Yang C Liu Y Lin Z. Large tumor size is a poor prognostic factor of gastric cancer with signet ring cell: Results from the surveillance, epidemiology, and end results database. Medicine (2019) 98(40):e17367. 10.1097/MD.0000000000017367 31577736 PMC6783183

[32] Chon HJ Hyung WJ Kim C Park S Kim JH Park CH. Differential prognostic implications of gastric signet ring cell carcinoma: stage adjusted analysis from a single high-volume center in Asia. Ann Surg (2017) 265(5):946. 10.1097/SLA.0000000000001793 27232252 PMC5389602

[33] Zhang M Zhu G Zhang H Gao H Xue Y. Clinicopathologic features of gastric carcinoma with signet ring cell histology. J Gastrointest Surg (2010) 14(4):601–6. 10.1007/s11605-009-1127-9 20033340

[34] Honoré C Goéré D Messager M Souadka A Dumont F Piessen G. Risk factors of peritoneal recurrence in eso-gastric signet ring cell adenocarcinoma: results of a multicentre retrospective study. Eur J Surg Oncol (2013) 39(3):235–41. 10.1016/j.ejso.2012.12.013 23313257

